# *QuickStats:* Age-Adjusted Percentage* of Adults Aged ≥18 Years Reporting a Lot of Pain,**^†^** Among Those Who Report Pain on at Least Some Days in the Past 3 Months,**^§^** by Poverty Status**^¶^** and Frequency of Pain — National Health Interview Survey, 2016–2017**

**DOI:** 10.15585/mmwr.mm6819a7

**Published:** 2019-05-17

**Figure:**
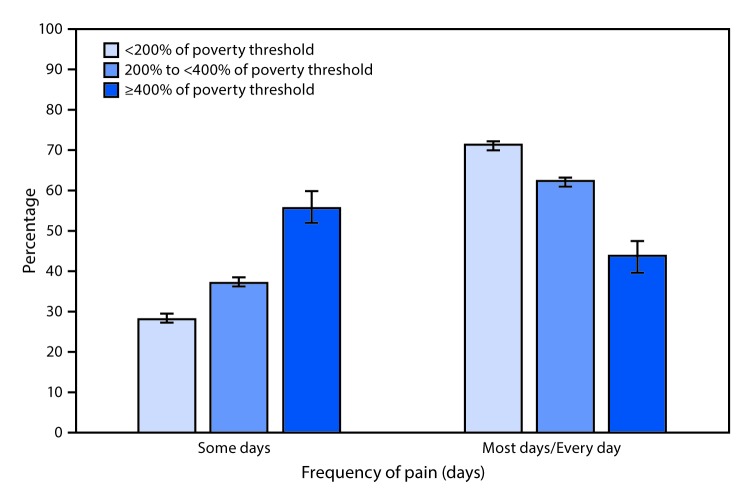
During 2016–2017, among those reporting pain, the percentage of adults ≥18 years who experienced a lot of pain on some days in the last 3 months increased with family income, from 28.6% among those with income <200% of the poverty threshold to 55.9% among those with income ≥400% of the poverty threshold. In contrast, the percentage reporting a lot of pain on most or every day decreased with increasing family income, from 71.4% among those at the lowest income level to 44.1% among those at the highest income level.

**Source:** National Health Interview Survey, 2016–2017. https://www.cdc.gov/nchs/nhis/index.htm.

